# The Bioactivities of Phycocyanobilin from Spirulina

**DOI:** 10.1155/2022/4008991

**Published:** 2022-06-11

**Authors:** Yi Li

**Affiliations:** College of Bioscience and Biotechnology, Hunan Agricultural University, Hunan Provincial Engineering Research Center of Applied Microbial Resources Development for Livestock and Poultry, Changsha, Hunan 410125, China

## Abstract

Phycocyanobilin (PCB) is a linear open-chain tetrapyrrole chromophore that captures and senses light and a variety of biological activities, such as anti-oxidation, anti-cancer, and anti-inflammatory. In this paper, the biological activities of PCB are reviewed, and the related mechanism of PCB and its latest application in disease treatment are introduced. PCB can resist oxidation by scavenging free radicals, inhibiting the activity of nicotinamide adenine dinucleotide phosphate (NADPH) oxidase, and delaying the activity of antioxidant enzymes. In addition, PCB can also be used as an excellent anti-inflammatory agent to reduce the proinflammatory factors IL-6 and IFN-*γ* and to up-regulate the production of anti-inflammatory cytokine IL-10 by inhibiting the inflammatory signal pathways NF-*κ*B and mitogen-activated protein kinase (MAPK). Due to the above biological activities of phycocyanobilin PCB, it is expected to become a new effective drug for treating various diseases, such as COVID-19 complications, atherosclerosis, multiple sclerosis (MS), and ischaemic stroke (IS).

## 1. Introduction

Phycobiliprotein, which is found in cyanobacteria and red algae, is a unique photosynthetic light-harvesting antenna. The most common phycobiliproteins in cyanobacteria are C-phycocyanin, allophycocyanin, and phycoerythrin. They all contain covalently bonded chromophore phycocyanobilin (PCB), giving them different absorption and emission spectra in the visible range [1]. PCB is a pigment protein essential for capturing and sensing light in phycocyanin (PC). As an effective antioxidant, anti-inflammatory agent, and anti-cancer agent, PC has been used to treat a variety of diseases by scavenging reactive oxygen species, such as cataracts, nonalcoholic fatty liver, and degenerative diseases (Table 1). The beneficial biological activities of PC are attributed to the covalently linked chromophore PCB [2], which usually covalently binds to the apolipoprotein of PC and is part of the biological functions of PC.

There are some ways to obtain PCB. For example, PCB can be obtained by reducing biliverdin (BV) catalysed by ferredoxin oxidoreductase (Pcya). Two enzymes play a vital role in the production of PCB by a two-step enzyme reaction: ferredoxin-dependent heme oxygenase (Ho1) and phycocyanin: ferredoxin oxidoreductase (Pcya) [3, 4]. Ho1 catalyses the ring-opening reaction of cyclic tetrapyrrole heme and promotes the chain opening of tetrapyrrole biliverdin IX *α* (BV), and Pcya further reduces it to PCB [5]. Using this method, PCB can be extracted and separated directly from Spirulina platensis. In addition, the key enzyme gene of PCB can be cloned into *E. coli*, and the biosynthesis is realised with autologous heme as a substrate [5–7]. As early as 20 years ago, some scholars successfully expressed *Ho1* and *Pcya* genes in Synechocystis, together with PCB from the same organism, and holophytochome was produced in *E. coli* [6]. Some scholars have also successfully created recombinant PCB in fermentation bottles by optimising induction time, culture medium, and other culture conditions, and the yield has been greatly improved [7].

PCB has anti-tumour, antioxidant, and anti-inflammatory functions, is a potential natural anti-inflammatory agent, and has a wide range of pharmaceutical values. As an excellent antioxidant, anti-inflammatory, and anti-cancer agent, PCB is widely used in medicine, cosmetics, food industry, medical care, and other fields and has significant commercial value (Table 1) [8–10]. With a variety of biological activities, PCB can remove damaged nerve cell-free radicals and avoid DNA oxidative damage to prevent nerve cell apoptosis caused by free radicals [8].

In addition, PCB can induce cytotoxicity and immune stimulation, promote the regeneration of animal blood cells, improve lymphocyte activity, improve the body's immune function, and comprehensively improve the body's disease resistance [9]. Studies have shown that PCB inhibits the activity of nicotinamide adenine dinucleotide phosphate (NADPH) oxidase [10], thus helping to prevent and treat various diseases caused by NADPH oxidase (NOX) in affected tissues. PCB also inhibits the proliferation of inflammatory cells and the production of inflammatory factors [11].

PCB has also been widely used in disease treatment. Studies have shown that PCB can reverse CCl_4_ induced liver injury in mice through anti-inflammatory ability [11], prevent diabetes nephropathy, protect nerves, improve ischaemic stroke (IS) by inhibiting oxidative stress, and cure atherosclerosis by activating HMOX1 [12–14]. Some studies have also shown that PCB can inhibit a variety of tumours, such as breast cancer [12] and liver cancer [13].

This paper reviews the bioactivity of CPB, its related mechanisms, and its application in disease treatment.

### 1.1. Antioxidant Capacity

Reactive oxygen species (ROS) are naturally oxygen-derived molecules produced by the human body, typically a by-product of intercellular metabolism with functions in host defence, immune regulation, and cell proliferation or differentiation; most members are different radicals [29, 30]. In our bodies, these free radicals can react with organic molecules, such as lipids, proteins, and DNA [31]. When the ROS concentration is low, it is beneficial to the organisms. However, the massive accumulation of ROS probably induces several diseases [32]. Oxidative stress occurs, producing a series of toxic metabolites in the body. For example, lipid peroxidation causes the toxic end product malondialdehyde (MDA), which can induce essential macromolecules, such as proteins and nucleic acids, to crosslink polymerisation, resulting in cytotoxicity.

Some investigations indicated that ROS would destroy the balance between the antioxidant defence system and free radicals and cause oxidative stress that damages DNA, proteins, and lipids [33, 34]. In a normal situation, our body's antioxidant defence system can generate antioxidant compounds to remove surplus ROS to guarantee intercellular metabolism functions well [30, 35]. However, in some diseases, the body's ability to scavenge ROS is weakened, and oxidants need to be used for intervention treatment. Compared with oxidants, such as special proteins and peptides, natural polysaccharides, ascorbic acid, carotenoids, glutathione, and phenolic compounds [36–38], and antioxidant compounds, such as Trolox, ascorbic acid, and reduced glutathione [14], PCB is the most effective antioxidant with a high oxygen radical absorbance capacity (ORAC).

NADPH oxidase (NOX) is the primary source of ROS because the overactivation of NOX causes the accumulation of ROS [39]. PCB can decrease diabetic vascular complications by degrading NOX and normalising urinary and renal oxidative stress markers [15, 24, 40]. In the treatment of acute liver failure (ALF), PCB decreases injuries to the liver structure under CCl_4_ and protects the proliferation of liver cells with antioxidants [41]. Moreover, PCB can also prevent oxidative stress and kidney damage caused by HgCl_2_ [42]. PCB can activate heme oxygenase-1 (Hmox1), the critical enzyme in the heme catabolic pathway responsible for forming the effective antioxidant bilirubin. Furthermore, PCB can regulate important markers of oxidative stress and endothelial dysfunction, such as eNOS, p22 NOX subunit, and vascular cell adhesion molecule-1 (VCAM-1) [25]. In addition, PCB can promote the biosynthesis of glutathione, a traditional antioxidant, and increase the expression and activity of various antioxidant enzymes [26].

Multiple studies revealed that PCB plays a crucial role in treating many notable neurodegenerative diseases, including Parkinson's and Alzheimer's, by reducing oxidative stress levels [43–45]. Parkinson's disease (PD) results primarily from the death of dopaminergic neurons in the substantia nigra. Clinical characteristics include tremors at rest, rigidity, slowness, lack of voluntary movement, and postural instability freezing, which significantly interfere with patients' daily lives [46]. When the antioxidant ability of cells decreases, free radicals can cause severe damage and even death to dopamine-produced cells. This harmful compound is usually accompanied by the activation of NOX, mitochondrial dysfunction, and hydrogen peroxide (H₂O₂) decomposition [47]. Oxidative stress is a common pathological mechanism in PD pathology. It induces mitochondrial dysfunction, mitochondrial DNA deficiency, pathological mutations, and changes mitochondrial morphology. The association of pathogenic proteins with mitochondria leads to the death of neurons [48, 49]. Therefore, decreasing oxidative stress levels is an effective treatment for PD patients and is also the PD-curing mechanism of PCB.

In addition, oxidative stress is also an important culprit in Alzheimer's disease (AD). AD is a disease that elicits the degeneration of cells and tissue in the brain and is the leading cause of dementia. Generally, the disorder's symptoms start with mild memory obstacles and grow into cognitive damage, dysfunctions in a complicated daily routine, and several other aspects of cognition [50].

Regarding the pathogenesis of AD, two primary hypotheses were elucidated: (1) cholinergic and (2) amyloid [51, 52]. The sedimentation of the amyloid *β* protein (A*β*P), the principal constituent of the plaques, is the inducing compound of AD pathology and directly results in neurofibrillary tangles, cell loss, vascularisation damage, and dementia [53]. Brain is easier to get stuck in oxidative stress than in other organs due to its consumption—20% of the oxygen provided by the respiratory system [54]. Further, most of the constituent neurons, such as lipids, proteins, and nucleic acids, can be oxidised in AD via several methods: mitochondrial dysfunction, enhanced metal levels, inflammation, and *β*-amyloid (A*β*) peptides.

Moreover, oxidative stress contributes to the progression of AD by facilitating A*β* sedimentation, tau hyperphosphorylation, and the loss of synapses and neurons [55]. The link between oxidative stress and AD indicates that oxidative stress is a pivotal part of pathological development and that antioxidants, such as PCB, may be effective in AD treatment [55].

More surprisingly, thanks to the inhibitory effect of PCB on NOX activity and aryl hydrocarbon receptor (AhR) agonism [2], PCB is suggested as a potential nutraceutical method to reverse nervous system injury induced by COVID-19.

### 1.2. Anti-Inflammatory Capacity

Inflammation is one of the organism's most fundamental and prominent protective reactions [56]; it is an adaptive response induced in many ways, such as microorganism invasion and tissue damage [57]. Hence, the inflammation process can be mild, such as tissue necrosis, or violently destroy organs, and contribute to a wide variety of physiological and pathological processes, such as cancer, acute interstitial nephritis, meningitis, arthritis, asthma, atherosclerosis, autoimmune diseases, diabetes, cancer, and conditions related to ageing [58].

Early in 1998, a study found that cytokines and chemokines were the primary mediators of the inflammation process [58]. In addition, multiple natural plant extracts or bioactive compounds predominantly affect the inhibition of inflammation by blocking major signalling pathways, such as NF-*κ*B and mitogen-activated protein kinases (MAPKs), which play a central role in producing numerous proinflammatory mediators [59]. Free radicals and ROS are critical mediators in activating inflammatory signalling pathways, such as NF-*κ*B and MAPK. Generally, the concentration of ROS and free radicals maintains a normal level that cannot activate inflammatory signalling pathways. In contrast, once ROS and free radical levels are significantly elevated, the inflammatory signalling pathway is activated immediately, and inflammation occurs. PCB can remove free radicals and reduce ROS by reducing the activation of NOX.

Some researchers have suggested that PCB reduces the expression levels of proinflammatory factors, such as IL-6 and IFN-*γ* to avoid inflammation [28, 45]. Moreover, PCB can also act as an AhR agonist to promote the transcription of genes encoding NrF-2 and up-regulate phase 2 induction of antioxidant enzymes, such as HO-1. HO-1 is a cell-protective molecule regulated by NrF-2, which can play a crucial role in anti-inflammation due to multiple adjusting methods. A study has proven that HO-1 can inhibit toll-like receptor 4 (TLR4)-dependent proinflammatory cytokine generation in activated macrophages [60] and inhibit the production of TNF-*α*, IL-1*β*, IL-6, and MIP-1*β*, which is induced by lipopolysaccharide (LPS) [61]. On the other hand, HO-1 can promote the production of the anti-inflammatory cytokine IL-10 [62]. Furthermore, PCB may mimic biliverdin to activate biliverdin reductase-mediated anti-inflammatory signalling pathway Akt to increase the IL-10 level, achieving the goal of anti-inflammation [2, 63].

The anti-inflammatory capacity of PCB is also related to inhibiting the expression of inflammation-related genes and suppressing the cytotoxicity of microglia in some neurodegenerative diseases [64]. Microglial cells play a crucial role in host defence and tissue recovery in the central nervous system. Under pathological circumstances, activated microglia are responsible for neuronal injury induced by inflammation [65]. Plenty of proinflammatory cytokines, chemokines, and inflammatory mediators are released by activated microglial cells, such as tumour necrosis factor TNF-*α*, interleukin IL-1, IL-6, and IL-8, macrophage inflammatory protein MIP-1*α* and MIP-1*β*, and ROS [66, 67]. Microglia can also induce severe brain impairment in several neurodegenerative disorders, such as AD and PD [68, 69].

### 1.3. Anticancer Capacity

Cancer is one of the most dreaded diseases globally and is the leading cause of death [70]. Researchers have attempted various approaches to cure cancer in recent decades. Examples include gene therapy and cancer vaccines, and the most common are surgery, chemotherapy, and radiotherapy [71–73]. Nevertheless, some effectively cure earlier-stage cancer but do not favour the advanced or recurrent stages. Other therapies are not considered practical enough due to their short history and immature technique [71, 74]. Moreover, in recent years, natural extracts from plants have been discovered and developed as a new treatment. Examples include green algae, cyanobacteria, and fungi [75–77].

Many articles have pointed out the anti-cancer or mitigating abilities of PCB [19]. Specifically, PCB suppresses cancer through its anti-proliferative effects on cancer cells [20]. PC, a type of pigment containing and utilising PCB as a functional chromophore, plays a crucial role in inducing cell apoptosis and cell cycle arrest and preventing cell migration and colony formation [21, 22]. More specifically, a study of breast cancer discovered that PC exerts an anticancer effect via the MAPK signalling pathway in cells named MDA-MB-231, a fundamental breast cancer cell. The MAPK pathway is pivotal in the promotion and progression of cancer [78], including cell proliferation, senescence, differentiation, migration, and apoptosis [79–81]. The apoptosis induced by PC is regulated by suppressing the extracellular signal-regulated kinase (ERK) pathway and the activation of the c-Jun N-terminal kinase (JNK) pathway and p38 MAPK pathways. These cascades are associated with cancer progression. Many studies have proven that JNK can activate caspases and regulate apoptosis-associated proteins. Furthermore, p38 MAPK can facilitate cell demise or increase cell development and survival, and activated ERK can influence the pathogenesis and progression of cancer.

Moreover, PC can elicit the block of the G0/G1 cell cycle by downgrading the expression of cyclin D1 and CDK2 in MDAMB-231 cells and upgrading the protein expression levels of p21 and p27. Moreover, PC only exerts a toxic effect on cancer cells, and it is safe for normal organisms. As a function group, PCB embodies these characteristics in the same way [12, 82].

In addition, PCB can reduce cancer risk by suppressing oxidative stress, which is one of the most crucial factors in cancer formation. Oxidative stress is a cell state induced by surplus ROS. When the ROS level overrides the antioxidant defence mechanisms, it can damage genomic and mitochondria DNA and cause molecule mutation, signalling pathways to change. Moreover, as one of the most powerful ROS family members, hydroxyl radical attacks DNA molecules and produces an adduct, 8-OH deoxyguanosine (8-OHdG), which is directly related to mutation enhancement, such as altering GC pair to TA pair in DNA replication [83, 84]. As a result of high-level ROS, oxidative stress can have a more serious and profound influence. It puts the cell in a sensitive environment, making it easier to get oxidation activated for some key signalling pathways or factors, such as MAPK and NF-*κ*B. NF-*κ*B is a nuclear transcription factor that participates in normal cell progress, such as inflammation regulation and cell growth [85]. In a normal situation, NF-*κ*B combines with NF-kappa-B inhibitor (IkB) to maintain a non-activated state. Still, some stimuli, including oxidative stress, can activate NF-*κ*B to promote its transport to nuclear and participate in gene expression [86]. The activated NF-*κ*B is associated with cancer by promoting tumour cell survival, vessel formation, and transportation. Oxidative stress enhancement can also alter the cell's redox potential to regulate gene expression [87].

According to various studies, PCB has been confirmed as a powerful antioxidant to eliminate ROS and reduce oxidative stress, a potential way to treat cancer.

### 1.4. The Role and Application of PCB in Disease Treatment

One study demonstrated the ability of PCB to inhibit diabetes, tested in a rodent model via oral administration [23]. Oxidative stress can result in the decoupling of endothelial nitric oxide synthase (eNOS) and the loss of nitric oxide (NO) bioactivity, namely the essential mediator of the vascular and microvascular complications of diabetes [24]. Many investigations have shown that oxidative stress is typically elicited by increasing NOX activation [24, 88]. PCB was administrated as a powerful agent to suppress NOX activation and has been proven to protect mice with diabetes under glomerulosclerosis [24]. Inhibition of NOX is a crucial bioactivity of PCB. This process enables PCB to act as an agent to restrain cardiac remodelling in mice suffering from myocardial infarction, aortic constriction, or rapid atrial pacing. It also avoids hepatic fibrosis by inhibiting proliferation and activation of hepatic stellate cells and abating the neurotoxic impact of temporary cerebral ischaemia [10, 45, 89, 90].

Hence, the development of PCB-associated healthcare food supplements may be a complex, but effective nutrition therapy. One study investigated the protective effect of PCB in atherosclerosis [25]. In addition, PCB is a medical agent used to modulate atheroprotective heme oxygenase-1 (Hmox1), an essential enzyme to protect against atherosclerosis through active heme catabolic pathways, and an upgraded expression of Hmox1 in atherosclerotic aortic lesions of rats was presented after feeding with PCB. Furthermore, PCB can regulate essential makers of oxidative stress and dysfunction of the endothelium, for instance, eNOS, p22 NOX subunit, and VCAM-1, representing an important position in treating atherosclerosis [25].

Asthma is caused by chronic inflammation of the lower respiratory tract. More specifically, NOX affects multiple levels of asthma pathogenesis, such as the proliferation of the smooth airway muscle and facilitating hypercontractility and hypertrophy. This allows lung influx of eosinophils through VCAM-1 and regulates mast cell activation elicited by allergens [26, 91]. A few studies have shown that PCB has the potent capacity of anti-asthma by inhibiting NOX [26].

Bhat and Madyastha demonstrated that PCB is important in reducing oxidative damage in DNA, which is induced by a mediator, peroxynitrite (ONOO(-)); PCB is effective in removing ONOO- [34]. In addition, PCB can inhibit the harmful biological effects mediated by ONOO- and manifest a potential therapeutic compound. Furthermore, a long period of PCB application can reduce the hazard of stroke due to the protective bioefficacy of the nitric oxide generated by cerebrovascular endothelium and antihypertensive efficacy [27]. More specifically, PCB therapy prevented the H_2_O_2_ and glutamate-caused PC12 cell injury and modulated 190 genes related to several immunological and inflammatory processes in BCCAo rats. Because the inflammatory process and oxidative stress are related to the stroke cascade, these results demonstrate the potential of PCB as a stroke medicine [9].

In addition, PCB has become a new choice for treating IS patients due to its neuroprotective effects observed in experimental ischaemic strokes [92]. Compared with bilirubin, a potent bioactive agent, PCB is safe for its source. Spirulina platensis is generally recognised as safe (GRAS) by the US Food and Drug Administration [93]. Bilirubin must be considered carefully due to its neurotoxicity risks resulting from overload [94]. Myelin loss exerts an essential impact on multiple sclerosis (MS) and ischaemic stroke (IS). Although many MS treatments are approved, none of them facilitate remyelination in patients, restricting their capacity for chronic recovery [95]. PCB has a positive effect on resolving inflammation and myelination and improving neuroinflammation, protecting against demyelination and axonal loss. PCB use would also modulate the expression of the anti-inflammation gene to maintain a homeostatic environment for the body [9, 28].

Because of the failure to form or preserve tight conjunctions, intestinal barrier function may be damaged or lost. The resulting inflammation may activate MAPKs. It has been proven that cellular Src (c-Src) induces the dissociation of tight junctions that can inhibit the harmful effect of overactivated MAPK and c-Src on the intestinal barrier [96].

PCB was also suspected of having a clinical potential for curing human autoimmune or allergic syndromes via enhancing regulatory T cells (Treg) activity. [97] Treg cells are critical to peripheral immune tolerance and the prevention of autoimmunity and tissue damage [98].

### 1.5. Summary and Prospect

Our research shows that PCB is a powerful and potential agent with plenty of bioactivities, antioxidants, anti-inflammatories, and anti-cancer ingredients. Regarding antioxidants, PCB achieves this goal in several ways: scavenging free radicals and ROS, delaying NOX activity, and up-regulating the activity of antioxidant enzymes. In PCB, multiple mechanisms exist inhibiting the inflammatory signalling pathways, such as NF-*κ*B and MAPK, by reducing the mediator ROS, suppressing the expression levels of proinflammatory factors IL-6 and IFN-*γ*, and up-regulating the production of anti-inflammatory cytokines IL-10. PCB induces the apoptosis of cancer cells under the suppression of the ERK pathway and the activation of the JNK and p38 MAPK pathways. It further elicits the block of G0/G1 cell cycles by downgrading the expression of cyclin D1 and CDK2 in cells and upgrading the protein expression level of p21 and p27.

PCB is a novel method to treat various diseases, such as COVID-19, cancer, atherosclerosis, diabetic nephropathy, MS, and IS. However, research on PCB is not perfect. The elaboration of antioxidant, anti-inflammation, and anticancer benefits is just the tip of the iceberg. Without more data, our understanding of PCB mechanisms for curing diseases is not precise. Hopefully, these mechanisms will be clarified in the future, and PCB can be an effective drug for curing more diseases.

## Figures and Tables

**Table 1:** The bioactivities of phycocyanobilin and phycocyanin.

Bioactivities	Bioactive compound	Mechanism of action	Reference
Antioxidant	Phycocyanobilin	Scavenge ROS and radicals	[14]
	Phycocyanin	Scavenge ROS and radicals	[15, 16]
Anti-inflammation	Phycocyanobilin	Inhibit the expression of inflammation-related genes Downregulate the quantity level of proinflammatory mediator	[15, 17, 18]
Anticancer	Phycocyanobilin	Antiproliferative effect on cancer cells	[19, 20]
	Phycocyanin	Induce cell apoptosis and cell cycle arrest Prevent cell migration and colony formation	[21, 22]
Other applications			
Diabetes	Phycocyanobilin	Suppress NADPH oxidase activation	[23, 24]
Atherosclerosis	Phycocyanobilin	Modulate atheroprotective heme oxygenase-1 (Hmox1) Regulate essential makers of oxidative stress	[25]
Asthma	Phycocyanobilin	Inhibit NADPH oxidase	[26]
Stroke	Phycocyanobilin	Protect vascular endothelium	[9, 27]
Multiple sclerosis (MS)	Phycocyanobilin	Protect demyelination and axonal loss	[9, 28]

## Data Availability

This review does not contain any original data.
